# Safety and Feasibility of Repeated Intrathecal Allogeneic Bone Marrow-Derived Mesenchymal Stromal Cells in Patients with Neurological Diseases

**DOI:** 10.1155/2019/8421281

**Published:** 2019-07-25

**Authors:** Kuang Pan, Lingna Deng, Peiying Chen, Qingxia Peng, Jingrui Pan, Yanfeng Wu, Yidong Wang

**Affiliations:** ^1^Department of Neurology, Sun Yat-sen Memorial Hospital, Sun Yat-sen University, Guangzhou 510120, China; ^2^Scientific Research Centre and Department of Orthopaedics, The Seventh Affiliated Hospital, Sun Yat-sen University, Shenzhen 518107, China; ^3^Center for Biotherapy, Sun Yat-sen Memorial Hospital, Sun Yat-sen University, Guangzhou 510120, China; ^4^Guangdong Province Key Laboratory of Brain Function and Disease, Zhongshan School of Medicine, Sun Yat-sen University, Guangzhou 510080, China; ^5^Guangdong Provincial Key Laboratory of Malignant Tumour Epigenetics and Gene Regulation, Sun Yat-sen Memorial Hospital, Sun Yat-sen University, Guangzhou 510120, China

## Abstract

Mesenchymal stromal cells (MSCs) have become the most commonly used adult stem cells in regenerative medicine. Preclinical studies have shown that MSCs-based therapy is a potential new treatment approach for neurological diseases. Intrathecal injection has unique feature which allows stem cells to directly migrate to the lesion site in patients with central nervous system (CNS) diseases. In this study, we evaluate the safety and feasibility of intrathecal allogeneic bone marrow-derived MSCs (BM-MSCs) in patients with neurological diseases. This open-label clinical study included 37 patients (14 diseases). Eligible patients underwent a baseline assessment and were intrathecally injected with allogeneic BM-MSCs (1 × 10^6^ cells/kg, 4 consecutive treatments at 1-week intervals). After four infusions, the patients were followed up for at least 6 months. Adverse events, cerebrospinal fluid (CSF) test results, clinical symptoms, physical examination, and haematological and imaging examinations were used to assess the safety and feasibility of the treatment. Also, we performed a systematic review of the safety of all types of intrathecal stem cells and compared our result to previous studies. In our study, the highest adverse event was a slight ache at the injection site (4.11%), followed by fever (3.42%) and mild headache (2.05%). No severe adverse events were reported. After the intrathecal injections, the white blood cell (WBC) counts in the CSF increased in 30 patients and the protein concentration in the CSF exceeded the normal range in 26 patients, while other CSF indicators remained normal. Moreover, these patients had no suspected manifestations of CNS infection. Haematological and imaging examinations showed no abnormal changes after BM-MSCs infusion. Compared with previous studies, the incidence of adverse events was nearly consistent or even lower for headache, fever, nausea, and neck pain. In conclusion, repeated intrathecal allogeneic BM-MSCs are safe, feasible, and promising for the treatment of patients with neurological diseases.

## 1. Introduction

Currently, many neurological diseases are difficult to cure and may even gradually progress after treatment. Patients with refractory neurological diseases suffer from paralysis, loss of social function, and difficulty of living, which places a heavy burden on society and their families. The promotion of neurological functional recovery and delaying disease progression are the main treatment goals in refractory neurological diseases [1]. Stem cells have the potential for self-renewal and multidirectional differentiation and are therefore ideal cell sources for nerve regeneration and repair [2]. In recent years, stem cells have become a very promising new direction for the treatment of refractory neurological diseases [3].

Mesenchymal stromal cells (MSCs) derived from multiple tissues such as bone marrow (BM), umbilical cord, peripheral blood, and adipose tissue and under standard culture conditions can differentiate into a variety of cells including bone, fat, cartilage, neurons, hepatocytes, and cardiocytes [4]. MSCs are a group of cells which must express CD105, CD73, and CD90 and lack expression of CD45, CD34, CD14 or CD11b, CD79a or CD19 and HLA-DR surface molecules [5]. According to current criteria, the isolation of MSCs produces heterogeneous, nonclonal cultures of stromal cells containing stem cells with different multipotential properties, committed progenitors, and differentiated cells [3]. Due to their unique features, MSCs have become the most commonly used adult stem cells in regenerative medicine [3]. Because they have lower immune suppression properties and immunogenicity compared with other cell types, the implantation of allogeneic MSCs may be more feasible and appropriate in the treatment of human diseases [6]. In particular, the use of allogeneic MSCs transplantation in patients with ischaemic stroke is more suitable than autologous MSCs transplantation [7]. In terms of the routine of transplantation, there are four principal methods to introduce cells into the body in patients with neurological diseases: intracerebral or intraspinal injection, intrathecal injection, intra-arterial injection, and intravenous injection [8–10]. In patients with central nervous system (CNS) diseases, intrathecal injection allows for higher concentrations of stem cells to migrate to the lesion site compared with intra-arterial or intravenous injection. Moreover, intrathecal injection is safer than either intracerebral injection or intraspinal injection. Therefore, MSCs transplantation via intrathecal injection may be the best route for stem cell therapy in patients with neurological disorders [7].

In this clinical study, we aimed to assess the safety and feasibility of repeated intrathecal allogeneic bone marrow-derived MSCs (BM-MSCs) injections in patients with CNS diseases. These diseases, including severe stroke, are primarily caused by the degeneration and/or death of neurons in the brain and/or spinal cord and currently lack effective treatments [11–13]. We described the adverse events, cerebrospinal fluid (CSF) test results, clinical symptoms, and haematological and imaging examination results in patients enrolled in our study and compared our results with those achieved in previous related studies reported in the literature. This information is provided for follow-up clinical trials.

## 2. Materials and Methods

### 2.1. Patient Enrolment

We performed an open-label clinical study in the Department of Neurology, Sun Yat-sen Memorial Hospital. The study was approved by the Ethics Committee of Sun Yat-sen Memorial Hospital, Sun Yat-sen University (Guangzhou, China). Part of the study has been registered in the Chinese Clinical Trial Registry (registration number: ChiCTR-INR-16008908). The inclusion criteria used for the study were as follows: (1) a neurological disease diagnosis that met the diagnostic criteria of Goldman's Cecil Medicine 24th edition [13] and (2) willingness of the patient and his/her family to sign an informed consent form and good compliance with examination, treatment, and follow-up. The exclusion criteria used for the study were as follows: (1) inflammatory or autoimmune diseases within half a year before recruitment (such as infectious diseases, systemic lupus erythematosus, rheumatoid or rheumatic disease, or thyroid disease); (2) glucocorticoid, immunosuppressant, or gamma globulin use within 15 days; (3) nosocomial infection; (4) a severe medical condition, such as cerebral hernia, status epilepticus, single or multiple organ failure, or unstable vital signs; (5) acute myocardial infarction; (6) hematologic disorders; (7) tumour; (8) pregnant or lactating women; (9) allergy to local anaesthetic; and (10) current participation in another clinical trial or participation in another clinical trial within 30 days.

### 2.2. Preparation of Allogeneic BM-MSCs

All procedures were performed at the Centre for Biotherapy (GMP certificate number: 2015A10413), Sun Yat-sen Memorial Hospital, Sun Yat-sen University (Guangzhou, China). All healthy donors were informed of the relevant scientific contributions of the study, the possible risks and complications of treatment, and the corresponding prevention and treatment measures for bone marrow aspirations. All participants then signed the informed consent form. The protocols for isolation, expansion, passaging, and storing of BM-MSCs were performed as described in our previous works [14, 15]. The procedures for preparing allogeneic BM-MSCs were provided in the Supplementary Materials (available here). After identifying MSCs immunophenotyping markers by flow cytometry, passages three to five were used for the clinical study.

### 2.3. Cell Therapy Protocol

Each patient received 4 consecutive allogeneic BM-MSCs treatments at 1-week intervals. Allogeneic BM-MSCs (1 × 10^6^ cells/kg body weight) in 10 ml normal saline were slowly infused intrathecally over approximately 10 min after the injection of a mixture containing 2 mg (0.4 ml) of dexamethasone and 0.6 ml of normal saline (to prevent aseptic chemical meningitis). After the infusion of the BM-MSCs, 2 ml of normal saline was injected to flush the syringe and spread the BM-MSCs.

### 2.4. Clinical and Laboratory Assessment

Basic information related to the patients was collected before BM-MSCs transplantation. Adverse events were monitored during the course of cell therapy and throughout follow-up. Routine, biochemistry and aetiological tests of CSF were performed at each injection. Clinical symptoms and physical examinations were performed at each injection and during follow-up. Haematological indicators (including blood cell counts and liver and renal function) were examined before and during BM-MSCs transplantation and in the first and the third month of follow-up. Chest X-ray, electrocardiogram, and magnetic resonance image (MRI) of the brain and spinal cord were checked before cell transplantation and in the twelfth month after transplantation. After four infusions, the patients were followed up for at least 6 months.

### 2.5. Review of the Literature

To increase our understanding of the safety of intrathecal injection of stem cells, we systematically reviewed the safety of relevant treatments in the literature. Our review included all types of stem cells administered via intrathecal injection in humans, and we compared the adverse events, CSF examinations, haematological indicators, and MRI results between these studies and our own study. We searched PubMed for all clinical trial articles published in English using the following search string: (“Injections, Spinal”[Mesh] OR “Spinal Puncture”[Mesh] OR “Subarachnoid Space”[Mesh]) AND (“Stem Cells”[Mesh] OR “Mesenchymal Stromal Cells”[Mesh]). We reviewed the bibliographies of retrieved articles. In the literature, stem cells must be injected into the subarachnoid space. Only reports with available clinical and biological data and outcomes were included. Intraspinal, intramedullary, and intracerebral injections were excluded from this review. Intrathecal injection associated with tumour treatment was also excluded. The relevant data about adverse events, CSF examinations, haematological indicators, and MRI results were collected to assess and compare the evaluations of the safety of intrathecal stem cells presented in previous studies with the data obtained in our study. In the summary of adverse events, we described the frequency of occurrences in terms of person-time to more accurately collect frequency data.

### 2.6. Data Analysis

The data are presented as the means ± SD or medians (range) for continuous variables and as a number (%) for qualitative variables. In the analysis of outcomes, ANOVA with Dunnett's multiple comparisons, Kruskal-Wallis tests, and chi-square tests were used where appropriate. All statistical tests were two tailed, and statistical significance was established at *P* < 0.05. All data were statistically analysed using SPSS 22.0 software (SPSS, Armonk, NY, USA), and all images were produced using GraphPad 7 software (GraphPad, San Diego, CA, USA).

## 3. Results

### 3.1. Baseline Characteristics of Patients in Our Study

From Dec. 2014 to Mar. 2018, a total of 14 diseases and 37 patients were evaluated (Table 1), including 12 cases of cerebral infarction, 5 cases of motor neuron disease, 4 cases of spinal cord injury, 3 cases of myelitis, 2 cases of spinocerebellar ataxia, 2 cases of multiple system atrophy, 2 cases of Alzheimer's disease, 1 case of acute disseminated encephalomyelitis, 1 case of encephalopathy syndrome, 1 case of hereditary spastic paraplegia, 1 case of intracerebral haemorrhage, 1 case of multiple sclerosis, 1 case of traumatic brain injury, and 1 case of thermoplegia.

The median age of the patients was 53 years old (range 18-75), and the median course of the diseases from the first injection was 8 months (range 0.4-120). In all, 25 men and 12 women were included. The median follow-up was 23 months (range 6-42).

### 3.2. Adverse Events in Our Study

The rate of mortality associated with BM-MSCs therapy during transplantation and follow-up was 0, although the following two patients died during the follow-up period for other causes: patient MND-022, who was a 75-year-old woman who died of respiratory failure due to an upper respiratory tract infection in the 11th month after BM-MSCs therapy, and patient MND-025, who was a 65-year-old man who died at home in the 30th month after therapy because of pneumonia. After a detailed inquiry, we concluded that these two deaths were not related to BM-MSCs therapy. No other severe adverse events, such as convulsions, condition aggravation or new neurological symptoms, transplantation, or tumourigenesis were discovered during the follow-up period in our study.

Six types of adverse events were observed in this study: headache, dizziness, fever, nausea, pain at the puncture site, and neck pain (Table 2). ADEM-003 had a fever after the first and second intrathecal injections, with both fevers occurring approximately 40 hours after injection. The first fever reached 37.9°C, and the second fever reached 38.2°C. After physical cooling and rehydration, the patient's body temperature dropped to normal. IS-015 had a fever on the day of the second intrathecal injection that reached a temperature of 40°C. The patient's body temperature returned to normal 3 h after oral administration of “Loxoprofen sodium tablets 60 mg”, and the fever did not recur. Patient SCA-030 felt chilly at 3 hours and had a fever at 18 hours after the first intrathecal injection. Her body temperature reached 39.2°C, and she was given a “1 ml diclofenac sodium” injection. After the injection, her body temperature returned to normal, and the fever did not recur. SCI-035 had a fever that reached 38.5°C at 12 hours after the first intrathecal injection; the fever was accompanied by diarrhoea, which was considered acute gastroenteritis. After receiving antibiotics, the fever did not recur. HSP-005 appeared to have pain in the forehead starting from 2 hours after the 4th intrathecal injection. The headache was aggravated when sitting and walking. At that time, low intracranial pressure was taken into consideration. This symptom gradually improved within 3-6 days after intensified intravenous fluid injection. MND-025 had moderate headache, nausea, no fever, no jet vomiting, no neck pain, and no signs of meningeal irritation on the day after the first and second intrathecal injections, and the patient's symptoms were relieved after rest. After 2 intrathecal injections, he gave up on stem cell therapy. AD-002 had mild neck pain beginning with the first intrathecal injection that lasted for approximately six months after the fourth injection. This pain was tolerable, and the patient was not given specific therapy. Other adverse effects included puncture site/lower back pain (6 person-time) and mild dizziness (2 person-time), both of which recovered on their own after lasting approximately 1-2 days.

### 3.3. CSF Examination in Our Study

**Figure 1:**
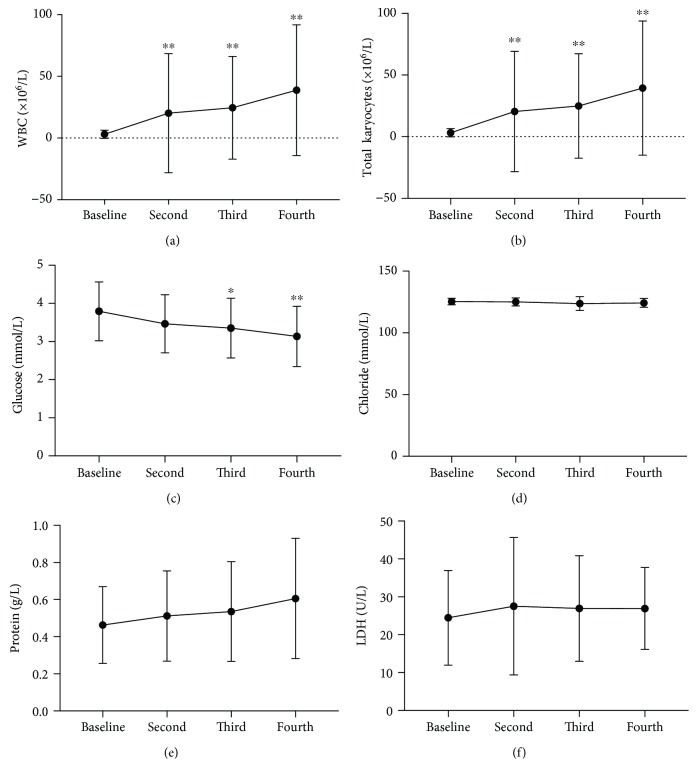
Routine and biochemical tests performed for the CSF. The trend of (a) WBC counts, (b) total karyocyte counts, (c) glucose, (d) chloride, (e) protein, and (f) LDH results in CSF. Statistical analysis was performed using one-way ANOVA with Dunnett's multiple comparisons. Results are presented as mean ± standard deviation. ^∗^*P* < 0.05; ^∗∗^*P* < 0.01.

The following routine biochemical examinations (Table 3) were performed: white blood cell (WBC) and total karyocyte counts, which were significantly higher in the treated patients (Figures 1(a) and 1(b)). Only 3 patients had WBC counts slightly higher than normal at baseline. After stem cell therapy, the WBC count increased in 30 patients, while seven patients maintained a normal WBC count. Changes in the total karyocyte counts were generally consistent with those observed for leukocytes. Glucose decreased, and the difference was statistically significant at the third and fourth examinations (Figure 1(c)), although the findings remained in the normal range. No significant changes in the chloride, protein, and lactate dehydrogenase (LDH) levels were observed (Figures 1(d)–1(f)). Before treatment, the protein concentrations exceeded the normal range (0.15-0.45 g/l) in 19 patients and were in the normal range in the other patients. After the intrathecal injections, the protein concentration exceeded the normal range in 26 patients, with the highest being 1.48 g/l. All patients had normal levels of chloride and LDH before and after transplantation. SCI-034 was a male patient with a spinal cord injury and high paraplegia. When the first and second lumbar punctures were performed, almost no CSF was available for collection. At the third and fourth lumbar punctures, his CSF was collected for examination. Therefore, he completed only two CSF examinations.

Regarding leukocytosis, we removed patients with erythrocytosis from the analysis because these patients had puncture damage at that time, which influenced the judgement of the CSF results. The remaining 21 patients (35 person-time) had leukocytosis. Among these patients, 8 (AD-001, HSP-005, ICH-006, IS-013, ML-021, MSA-028, SCA-030, and SCA-031) had a high WBC count (above 10 × 10^6^/l) (12 person-time) but a normal CSF biochemistry examination. HSP-005 appeared to have pain in the frontal area at the intrathecal injection site as described above. Fourteen patients (IS-007, IS-008, IS-009, IS-011, IS-014, IS-015, IS-017, ML-021, MND-022, MND-023, MND-025, MSA-029, SCI-032, and SCI-033) presented elevated WBC counts and protein levels (23 person-time). With the exception of IS-015, who had a fever after the second intrathecal injection, and MND-025, who had moderate headache and nausea after the second intrathecal injection, the remaining 12 patients had no suspected manifestations of CNS infection, such as headache, fever, or signs of meningeal irritation, during the treatment period and within the follow-up period. Moreover, the levels of glucose and chloride in the CSF were normal in all of these patients.

Every aetiological examination performed in the CSF of all patients was normal. Examinations for *Cryptococcus* were negative, and *Mycobacterium tuberculosis* was quantified at a level below 1.0 × 10^3^ copies/ml.

### 3.4. Haematological and Imaging Examinations Performed in Our Study

In our study, the haematological examination, chest X-ray, and electrocardiogram results showed no abnormal changes after BM-MSCs infusion. Twenty-two of the 30 patients with at least 12 months of follow-up underwent an MRI examination 12 months after stem cell therapy. The results showed no neoplasms within the cranial cavity and spinal canal.

### 3.5. Adverse Events in Intrathecal Autologous MSCs Clinical Studies in the Literature

Intrathecal autologous MSCs transplantations have been reported in humans (Table 4) primarily for amyotrophic lateral sclerosis [16–22], followed by multiple sclerosis [16, 23–26] and spinal cord injury [27–32]. In addition, this approach has been reported in traumatic brain injury [33], epilepsy [34], and cerebral palsy [35]. Three types of autologous MSCs have been included in these studies: BM-MSCs, peripheral blood MSCs, and adipose-derived MSCs. A combination of intrathecal and intravenous injections was also included for analysis. All dose statistics were divided into two injection modes: one mode used in eight studies was based on patient body weight, and the doses fluctuated between 0.1 × 10^6^/kg and 10 × 10^6^/kg with a median of 1 × 10^6^/kg; the other mode was based on the use of a constant dose for each injection related to the preparation of the stem cells, and the doses fluctuated between 0.7 × 10^6^ and 100 × 10^6^ with a median of 30 × 10^6^. The frequency of injection was also very variable, with some studies performing the injection once, and most studies performing 2-3 injections at intervals ranging from 5 days to one month. The follow-up periods ranged from 14 days to 826 days. A total of 518 patients (1028 person-time) were included in the evaluated intrathecal autologous MSCs clinical studies.

In previous studies, the most common adverse event was fever, which occurred in 7.88% (81/1028) of patients after treatment and was self-relieved or relieved after taking a drug. The next most common adverse event was pain at the injection site and back pain, which had an incidence rate of 7.30% (75/1028) and was related to the puncture operation. The occurrence of headache also attracted our attention because it occurred at a rate of 7.10% (73/1028) and might be related to changes in intracranial pressure. This symptom was alleviated by increasing hydration or was in some cases self-relieved. In addition, the proportions of patients with nausea and neck pain were 0.97% (10/1028) and 0.19% (2/1028), respectively. Other adverse events included adverse events in the motion system (including spasticity (5, 0.49%), difficulty walking/standing (4, 0.39%), weakness (3, 0.29%), rigidity (2, 0.19%), jerky movement (2, 0.19%), and neck stiffness (2, 0.19%)), adverse events in the sensory system (including leg and neuropathic pain (63, 6.13%) and tingling sensation (2, 0.19%)), and other events (including aseptic meningitis (49, 4.77%), vomiting (10, 0.97%), sweating (4, 0.39%), transient hypertension (4, 0.39%), urinary tract infection (4, 0.39%), bruising (3, 0.29%), dyspnoea (2, 0.19%), leukocytosis (2, 0.19%), confusion (1, 0.10%), syncope (1, 0.10%), nasopharingytis (1, 0.10%), and bronchitis (1, 0.10%)). Transient encephalopathy with seizures a few days after cell injection was reported in only one case [24]. In that case, the patient used intravenous valproate and recovered without significant sequelae. No other serious adverse events were reported.

The adverse events observed in our study and in previous studies of intrathecal autologous MSCs were compared (Table 5). The frequency of headache was significantly lower in our trial (*P* < 0.05), whereas the frequency of dizziness was higher in our trial because no dizziness was reported in previous autologous MSCs studies. No differences in fever, nausea, pain at the puncture site, and neck pain were observed between our study and previous studies.

### 3.6. Adverse Events in Intrathecal Allogeneic MSCs Clinical Studies in the Literature

Only four articles about intrathecal allogeneic MSCs clinical studies could be retrieved (Table 6). All MSCs in these studies were umbilical cord derived [36–39]. The infusion dose fluctuated between 1 × 10^6^ and 20 × 10^6^, and the median was 1 × 10^7^. The frequencies of injection were 2-12. The intervals ranged from 5 to 7 days. The follow-up periods were 3 to 36 months. A total of 67 patients (258 person-time) participated in these studies. The top three adverse events were headache in 2.71% (7/258), dizziness in 2.33% (6/258), and fever in 1.16% (3/258) of the cases.

The studies of intrathecal allogeneic MSCs only showed a lower ratio of pain at the puncture site, and this difference was significant when comparing with our results (Table 7). No differences in the other adverse events were observed between our study and previous studies.

### 3.7. Adverse Events in Intrathecal Non-MSCs Stem Cells in Clinical Studies in the Literature

To know about whether intrathecal MSCs were safer than intrathecal non-MSCs stem cells, we included clinical studies of intrathecal non-MSCs stem cells described in the literature and made a comparison. These non-MSCs stems cells included autologous bone marrow haematopoietic stem cells [40], autologous bone marrow mononuclear cells [35, 41–44], allogeneic cord blood mononuclear cells [38, 45], autologous bone marrow progenitor cells [46], autologous bone marrow aspirate concentrate [47], and autologous bone marrow stem cells [48]. Mononuclear cells and bone marrow aspirate concentrates contain some stem cells. Progenitor cells are a subpopulation of BM-derived haematopoietic stem cells. Therefore, these three types of cells were included in the analysis (Table 8).

Except for injection doses determined according to the unit weight, the doses in the other studies fluctuated between 1 × 10^6^ and 5387 × 10^6^, with a median of 4 × 10^7^. The frequency of injection was mostly once, and the most was 5 times. The interval ranged from 3 days to 1 month. The follow-up period was 1 to 24 months. These clinical studies included 604 patients (1241 person-time). The incidence of headache was the highest (8.94%, 111/1241), and it was followed by fever (8.78%, 109/1241). The rates of nausea and pain at the puncture site were 1.45% (18/1241) and 2.10% (26/1241), respectively. Other adverse events observed in intrathecal non-MSCs stem cells included events related to the motion system (including shivering (3, 0.24%) and spasm (2, 0.16%)) or the sensory system (including lower limb, muscle, and neuropathic pain (20, 1.61%) and tingling sensation (6, 5.48%)) and other events (including vomiting (20, 1.61%), lingual oedema (1, 0.08%), laryngeal stridor (1, 0.08%), and bronchoconstriction (1, 0.08%)). One serious adverse event was reported in a previous study [46]. A 9-year-old boy with a history of seizure stopped anticonvulsants 3 years before cell therapy, and seizures occurred once in the sixth month after cell therapy. The seizures were well controlled, and his examinations were normal.

Our results were significantly different from those reported in the previous literature of intrathecal non-MSCs stem cells with regard to headache, dizziness, and fever. The proportions of patients with headaches and fever were lower in our study than in previous studies. However, the rate of dizziness was slightly higher in our study (Table 9). This result is consistent with trials of autologous mesenchymal stem cells.

### 3.8. Comparison of Adverse Events among Clinical Studies of Intrathecal Autologous MSCs, Allogeneic MSCs, and Non-MSCs Stem Cells

We included our study in the analysis of allogeneic MSCs (Table 10). We found no significant difference in nausea and neck pain among these three groups, and the frequencies of all three were low. The proportion of patients with headaches and fever was lower in allogeneic MSCs than in autologous MSCs and other non-MSCs types. We unexpectedly found that dizziness occurred only in intrathecal allogeneic MSCs, although its occurrence rate was low. Autologous MSCs trials had the highest proportion of patients with pain at the puncture site. Moreover, as outlined above, other adverse events were reported in the literature in studies exploring intrathecal autologous MSCs and non-MSCs stem cells.

### 3.9. Other Safety Indicators Described in Previous Studies

Only a small number of studies contained information about examinations of CSF, haematological indicators, and MRI results. We analysed the 33 relevant articles and found that one literature detailed the results of CSF examinations, two articles listed results for haematology, and 18 articles described MRI results. In the study of CSF [21], nuclear cells and protein levels increased, while glucose levels slightly decreased but remained in the normal range. The literature containing data for haematological indicators showed that there were no significant changes [38, 45]. There were 18 articles [20–24, 26–28, 30–32, 36, 39, 42–44, 46, 48] that included MRI results obtained during follow-up, and no tumourigenesis was reported.

## 4. Discussion

To our knowledge, this clinical study is the first report to be published in English that describes repeated intrathecal injection of allogeneic BM-MSCs for the treatment of neurological diseases. A total of 37 cases were observed that involved 14 diseases with a maximum follow-up period of 42 months. We found that performing four intrathecal injections of allogeneic BM-MSCs at 1 × 10^6^ cells/kg body weight at an interval of one week was safe and produced no serious adverse events.

In our study, adverse events, clinical symptoms, physical signs, CSF tests, and haematological and imaging examinations were monitored. Adverse events were found in 37 patients and included puncture site/lower back pain (6 person-time), fever (5 person-time), headache (3 person-time), mild dizziness (2 person-time), nausea (2 person-time), and neck pain (1 person-time). Pain at the puncture site was associated with injury resulting from lumbar puncture and operator skill, and dizziness may be associated with changes in the volume of CSF. When manifestations such as fever, headache, and neck pain are encountered, CNS infection must be excluded. The WBC counts and protein concentrations of CSF were evaluated in the patients with these symptoms (ADEM-003, HSP-005, IS-015, MND-025, and SCA-030) and were found to be outside normal levels. However, the levels of glucose, chlorine, and LDH in the CSF were normal, and there were no signs of meningeal irritation. Therefore, CNS infection could be excluded, and the symptoms may be attributable to aseptic meningitis or low intracranial pressure. The CSF results of two patients (AD-002 and SCI-035) suffering adverse events were always in the normal range, indicating that their symptoms may have been attributable to acute infusion-associated toxicity. Cases were observed in which WBC counts and/or protein levels in CSF increased without adverse events. The causes of these CSF abnormalities may have been an acute response by intraspinal tissues to the BM-MSCs.

Compared with past clinical studies, adverse events in our study occurred at a greater or lower frequency. The frequencies of fever and headache were lower in our study than in those exploring non-MSCs stem cells, and the frequencies of headache were lower in autologous MSCs trials. The frequencies of nausea and neck pain were the same in this trial as in other MSCs trials. The proportion of patients with dizziness was slightly higher in our study than in those exploring autologous MSCs and non-MSCs stem cells, while the proportion with pain at the puncture site was slightly higher than those found in allogeneic MSCs trials. In those studies, other adverse events were reported; in particular, two serious adverse events were reported, and both were seizures. In the first article describing the occurrence of seizure [24], the author proposed that the event was attributable to the secondary lysis of high numbers of injected cells, especially because 50% of the cells were administered through a cisternal puncture. We suggest that the seizure might have been associated with the procedure used to prepare the cells, which was not described in detail. In the second article describing the occurrence of seizure [46], the authors stated that the patient had a medical history of seizure and experienced a reactivation of the prior epileptic seizures after the therapy. We suggest that the use of specific types of progenitor cells in children might stimulate epilepsy. We also summarized other safety indicators, such as examinations of CSF or haematological indicators and MRI results, and we found that there was no significant difference between the results reported in the literature and in our study.

We identified issues associated with collecting data on adverse events in previous clinical trials. First, some of the studies focused primarily on functional outcomes and did not record adverse events based on careful observation, and in some cases, the studies even ignored adverse events. Second, some studies did not record the data in an appropriate manner, with statistics in person-time being more suitable. Third, some of the trials observed adverse events within a short time period, which may have resulted in data loss. Fourth, when recording data in children and unconscious patients, some of the patients will not be able to fully express (or express at all) their feelings of discomfort. All these factors might result in inaccurate or incomplete data.

Certain deficiencies were observed in our study. First, because intrathecal injection is an invasive method which means that it is unethical for patients or healthy people to be intrathecally injected with placebo (such as normal saline), we did not set up a control group. It may be therefore difficult to exclude the placebo effect. Second, fewer cases were included; thus, bias might have been introduced in the trial. Lastly, the follow-up period was inconsistent and varied from 6 to 42 months. During the follow-up period, some patients did not undergo a review of MRI examinations, and it was therefore difficult to compare changes that occurred between timepoints before and after treatment.

After accessing the safety and feasibility of MSCs, we will further evaluate the effectiveness of intrathecal injection of MSCs in the treatment of specific neurological diseases. There have been many clinical trials and reviews to analyse the success rate of MSCs treatment. For applications of MSCs therapy, standardization procedures for MSCs production is the most critical step, rather than focusing on the clonality of MSCs [49]. Donor heterogeneity, ex vivo expansion, immunogenicity, and cryopreservation are important issues that must be addressed [50]. Evaluating the percentage of stem/progenitor cells before delivering MSCs to the patients is essential [51], while the presence of senescent cells in a batch of MSCs may also be taken into account [52]. More studies have shown that MSCs immunomodulatory activity has a lot to do with Indoleamine 2,3-Dioxygenase (IDO) production following treatment of MSCs with interferon gamma (IFN-*γ*) [53]. The consideration of these factors has far-reaching significance for the analysis of the effectiveness of repeated intrathecal allogeneic BM-MSCs in patients with neurological diseases.

## 5. Conclusions

In summary, the results of our study and the comparison of our data with the data presented in previous studies showed that intrathecal injection of allogeneic BM-MSCs for the treatment of neurological diseases is safe and feasible and has good clinical application prospects.

## Figures and Tables

**Table 1:** Clinical characteristics of the patients at the time of enrolment.

Case number	Type of disease	Age (years)	Gender	Onset time (months)	Intrathecal frequency (times)	Follow-up time (months)
AD-001	AD	69	F	120	4	12
AD-002	AD	54	M	36	4	9
ADEM-003	ADEM	38	F	3	4	6
ES-004	ES	24	F	54	4	6
HSP-005	HSP	40	M	120	4	12
ICH-006	ICH	19	M	9	4	23
IS-007	IS	52	F	3	4	42
IS-008	IS	44	M	36	4	39
IS-009	IS	72	M	0.8	4	38
IS-010	IS	73	F	1.5	4	37
IS-011	IS	44	F	2	4	12
IS-012	IS	66	M	1	4	37
IS-013	IS	36	M	8	4	12
IS-014	IS	62	M	0.8	4	12
IS-015	IS	64	M	1	4	29
IS-016	IS	53	M	1	4	26
IS-017	IS	55	M	1	4	25
IS-018	IS	70	F	0.4	4	9
ML-019	ML	20	M	1	4	24
ML-020	ML	31	M	1.5	4	12
ML-021	ML	18	M	6	4	25
MND-022	FAS	75	F	24	4	11
MND-023	ALS	42	M	24	4	12
MND-024	SMA	58	M	48	4	35
MND-025	PBP	64	M	12	2	30
MND-026	ALS	70	M	24	4	12
MS-027	MS	58	F	48	4	26
MSA-028	MSA	60	M	12	4	37
MSA-029	MSA	53	F	19	4	12
SCA-030	SCA	55	F	12	4	39
SCA-031	SCA	57	F	60	4	25
SCI-032	SCI	25	M	1	4	38
SCI-033	SCI	53	M	2	4	37
SCI-034	SCI	61	M	12	4	18
SCI-035	SCI	30	M	6	4	8
TBI-036	PTBS	30	M	36	4	18
TP-037	TP	33	M	2	4	9

Abbreviations: AD, Alzheimer's disease; ADEM, acute disseminated encephalomyelitis; ALS, amyotrophic lateral sclerosis; ES, encephalopathy syndrome; FAS, flail arm syndrome; HSP, hereditary spastic paraplegia; ICH, intracerebral haemorrhage; IS, ischaemia stroke; ML, myelitis; MND, motor neuron disease; MS, multiple sclerosis; MSA, multiple system atrophy; PBP, progressive bulbar paralysis; SCA, spinocerebellar ataxia; SCI, spinal cord injury; SMA, progressive spinal muscular atrophy; TBI, traumatic brain injury; TP, thermoplegia; M, male; F, female.

**Table 2:** Adverse events for intrathecal injection of allogeneic MSCs.

Adverse effects (no. of patients)	AD *N* = 2	ADEM *N* = 1	ES *N* = 1	HSP *N* = 1	ICH *N* = 1	IS *N* = 12	ML *N* = 3	MND *N* = 5	MS *N* = 1	MSA *N* = 2	SCA *N* = 2	SCI *N* = 4	TBI *N* = 1	TP *N* = 1	Total *N* = 37
Headache	0	0	0	1	0	0	0	2	0	0	0	0	0	0	3
Dizziness	0	0	0	0	0	1	1	0	0	0	0	0	0	0	2
Fever	0	2	0	0	0	1	0	0	0	0	1	1	0	0	5
Nausea	0	0	0	0	0	0	0	2	0	0	0	0	0	0	2
Pain at puncture site	0	0	0	0	0	3	0	1	0	1	1	0	0	0	6
Neck pain	1	0	0	0	0	0	0	0	0	0	0	0	0	0	1

Abbreviations: AD, Alzheimer's disease; ADEM, acute disseminated encephalomyelitis; ES, encephalopathy syndrome; HSP, hereditary spastic paraplegia; ICH, intracerebral haemorrhage; IS, ischaemia stroke; ML, myelitis; MND, motor neuron disease; MS, multiple sclerosis; MSA, multiple system atrophy; SCA, spinocerebellar ataxia; SCI, spinal cord injury; TBI, traumatic brain injury; TP, thermoplegia.

**Table 3:** Number of WBCs and levels of protein in the CSF in our study.

Patient	WBC (×10^6^/l)				Protein (g/l)			
	First	Second	Third	Fourth	First	Second	Third	Fourth
AD-001	0.3	8	18	12	0.24	0.28	0.27	0.26
AD-002	2	24	19	9	0.23	0.30	0.35	0.29
ADEM-003	0	1	1	0	0.54	0.58	0.69	1.10
ES-004	2	12	15	18	0.93	0.87	1.25	0.97
HSP-005	1	2	4	78	0.21	0.22	0.18	0.29
ICH-006	3	9	41	122	0.25	0.25	0.42	0.46
IS-007	6	6	20	26	0.66	0.64	0.81	0.62
IS-008	5	15	124	230	0.52	0.58	0.87	0.99
IS-009	1	2	12	3	0.46	0.58	0.53	0.51
IS-010	1	6	8	3	0.59	0.63	0.56	0.41
IS-011	3	11	9	10	0.54	0.50	0.49	0.53
IS-012	4	6	1	5	0.75	0.75	0.79	0.81
IS-013	2	35	36	25	0.26	0.35	0.30	0.31
IS-014	9	9	9	12	0.57	0.52	0.59	0.66
IS-015	2	13	33	45	0.55	0.70	1.26	0.85
IS-016	0	2	7	6.5	0.44	0.53	0.74	0.58
IS-017	3	7	6	11	0.69	1.04	0.40	1.35
IS-018	5	10	7	7	0.49	0.56	0.63	1.48
ML-019	2	1	2	/	0.52	0.35	0.26	/
ML-020	1	2	2	2	0.25	0.31	0.43	0.49
ML-021	12	16	19	72	0.25	0.29	0.29	0.58
MND-022	1	13	11	7	0.48	0.50	0.44	0.44
MND-023	1	12	11	18	0.60	0.76	0.54	0.46
MND-024	0	8	3	128	0.40	0.29	0.41	0.49
MND-025	2	15	/	/	0.35	0.48	/	/
MND-026	2	10	5	2	0.69	0.85	0.75	0.71
MS-027	2	2	4	0	0.31	0.34	0.37	0.35
MSA-028	2	4	5	18	0.26	0.28	0.25	0.34
MSA-029	1	112	206	155	0.24	0.55	0.42	0.50
SCA-030	2	13	17	23	0.26	0.22	0.29	0.31
SCA-031	3	19	23	81	0.21	0.23	0.24	0.26
SCI-032	6	26	49	49	0.64	0.59	0.59	0.99
SCI-033	15	280	119	112	0.80	1.19	0.98	0.95
SCI-034	/	/	0	16.4	/	/	0.55	0.49
SCI-035	4	3	4	11	0.34	0.33	0.32	0.20
TBI-036	2.6	7	21	34.3	0.24	0.22	0.28	0.23
TP-037	3	6	13	8	0.90	0.77	0.74	0.94

Abbreviations: AD, Alzheimer's disease; ADEM, acute disseminated encephalomyelitis; ES, encephalopathy syndrome; HSP, hereditary spastic paraplegia; ICH, intracerebral haemorrhage; IS, ischaemia stroke; ML, myelitis; MND, motor neuron disease; MS, multiple sclerosis; MSA, multiple system atrophy; PBP, progressive bulbar paralysis; SCA, spinocerebellar ataxia; SCI, spinal cord injury; TBI, traumatic brain injury; TP, thermoplegia.

**Table 4:** Clinical studies of intrathecal autologous MSCs described in the literature.

Authors/country	Diagnosis	No. of treated patients	Stem cell type	Transplant type	Dose per injection	Injection frequency	Follow-up	Adverse events (person-time)
Bonab et al. [23]/Iran	MS	10	Auto-BM-MSCs	IT	8.73 × 10^6^	1	13–26 months	Slight headache (9); iatrogenic meningitis (2)
Pal et al. [27]/India	SCI	30	Auto-BM-MSCs	IT	1 × 10^6^/kg	2–3, weekly	12–24 months	None
Karussis et al. [16]/Israel	MS ALS	15 19	Auto-BM-MSCs	IT+IV	63.2 × 10^6^ 54.7 × 10^6^	1	25 months	Fever (21); headache (15); meningism (1); rigidity (2); leg pain (3); dyspnoea (1); confusion (1); neck pain (1); difficulty walking/standing (4)
Yamout et al. [24]/Lebanon	MS	7	Auto-BM-MSCs	IT	(32‐100) × 10^6^	1	12 months	Seizures (1); cervical and lower back pain (3)
Kishk et al. [28]/Egypt	SCI	44	Auto-BM-MSCs	IT	(5‐10) × 10^6^/kg	6, monthly	12 months	Encephalomyelitis (1); neuropathic pain (24); sweating (3); transient hypertension (3); spasticity (4); dyspnoea (1); jerky movements (2)
Hammadi et al. [25]/Iraq	MS	50	Auto-PB MSCs	IT	(2‐7) × 10^6^	1–8 (mean 2.14)	12 months	Backache (45); meningism (45)
Bonab et al. [26]/Iran	MS	25	Auto-BM-MSCs	IT	29.5 × 10^6^	1	12 months	Fever (25); nausea/vomiting (2); headache (3); lower limb weakness (2)
Karamouzian et al. [29]/Iran	SCI	11	Auto-BM-MSCs	IT	(7‐12) × 10^5^	1	12–33 months	Pain (8)
Tian et al. [33]/China	TBI	97	Auto-BM-MSCs	IT	5 × 10^6^	1	2 weeks	Fever (5); headache (2)
Kim et al. [17]/Korea	ALS	37	Auto-BM-MSCs	IT	1 × 10^6^/kg	2, monthly	6 months	Fever (11); myalgia (9); lower back pain (4); headache (4)
Oh et al. [18]/Korea	ALS	8	Auto-BM-MSCs	IT	1 × 10^6^/kg	2, at 26-day intervals	12 months	Fever (4); administration site pain (3); headache (3)
Rushkevich et al. [19]/Belarus	ALS	10	Auto-BM-MSCs	IT+IV	(5‐9.7) × 10^6^	1–2, at 5–7-month intervals	12 months	Fever (1); headache (2)
Petrou et al. [20]/Israel	ALS	26	Auto-BM-MSCs	IT+IV	(1‐2) × 10^6^/kg	1	6 months	Headache (13); fever (11); back/leg pains (8); vomiting (3); neck stiffness (2); general weakness (1); bruising (1); spasticity (1)
Satti et al. [31]/Pakistan	SCI	9	Auto-BM-MSCs	IT	(0.9‐2.57) × 10^6^/kg	2–3, monthly	269–826 days	Headache (1); tingling sensation (2)
Hlebokazov et al. [34]/Belarus	EP	10	Auto-BM-MSCs	IT+IV	1 × 10^5^/kg	1	12 months	Headache (1)
Hur et al. [30]/Korea	SCI	14	Auto-AD MSCs	IT	3 × 10^7^	3, monthly	8 months	Urinary tract infection (1); headache (2); nausea and vomiting (1)
Staff et al. [21]/USA	ALS	27	Auto-AD MSCs	IT	(1‐10) × 10^7^	1–2, monthly	4–108 weeks	Headache (3); back/leg pain (9)
Liu et al. [35]/China	CP	33	Auto-BM-MSCs	IT	1 × 10^6^/kg	4, at 3–4-day intervals	12 months	Fever (2); nausea, vomiting, and headache (4)
Sykova et al. [22]/Czech	ALS	26	Auto-BM-MSCs	IT	(10.5‐19.5) × 10^6^	1	18 months	Headache (7); hyperhidrosis (1); leukocytosis (2)
Vaquero et al. [32]/Spain	SCI	10	Auto-BM-MSCs	IT	30 × 10^6^	4, 3 months	12 months	Hypertension (1); local pain (1); leg pain (1); urinary tract infection (3); headache (4); hyperthermia (1); wound (1); infected pressure ulcer (1); arthralgia (1); syncope (1); pain in coccyx (1); neck pain (1); back pain (1); nasopharingytis (1); bronchitis (1)

Abbreviations: AD, adipose derived; ALS, amyotrophic lateral sclerosis; auto, autologous; BM, bone marrow; CP, cerebral palsy; EP, epilepsy; MS, multiple sclerosis; MSCs, mesenchymal stem cells; PB, peripheral blood; SCI, spinal cord injury; TBI, traumatic brain injury; IT, intrathecal; IV, intravenous.

**Table 5:** Comparison of adverse events between our study and previous clinical studies of intrathecal autologous MSCs.

Adverse events (person-time)	Total (%) *N* = 1174	Our study (%) *N* = 146	Literature review (%) *N* = 1028	*P*
Headache	76 (6.47)	3 (2.05)	73 (7.10)	0.032
Dizziness	2 (0.17)	2 (1.37)	0 (0.00)	0.015
Fever	86 (7.32)	5 (3.42)	81 (7.88)	0.053
Nausea	12 (1.02)	2 (1.37)	10 (0.97)	0.995
Pain at puncture site	81 (6.90)	6 (4.11)	75 (7.30)	0.155
Neck pain	3 (0.26)	1 (0.68)	2 (0.19)	0.824

**Table 6:** Clinical studies of intrathecal allogeneic MSCs described in the literature.

Authors/country	Diagnosis	No. of treated patients	Stem cell type	Transplant type	Dose per injection	Injection frequency	Follow-up	Adverse event (person-time)
Jin et al. [36]/China	SCA	16	UCMSCs	IT+IV	2 × 10^7^	3, weekly	12 months	Fever (1); dizziness (2); headache (2)
Lv et al. [38]/China	Autism	9	UCMSCs	IT+IV	(1‐2) × 10^6^	2, at 5–7-day intervals	6 months	Fever (2)
Wang et al. [39]/China	TBI	20	UCMSCs	IT	1 × 10^7^	4, at 5–7 day intervals	6 months	Dizziness (4); headache (4)
Liu et al. [37]/China	SCI	22	UCMSCs	IT	1 × 10^6^/kg	4–12, weekly	3–36 months	Lumbago (1); headache (1)

Abbreviations: SCA, hereditary spinocerebellar ataxia; SCI, spinal cord injury; TBI, traumatic brain injury; UCMSCs, umbilical cord mesenchymal stem cells; IT, intrathecal; IV, intravenous.

**Table 7:** Comparison of adverse events between our study and previous clinical studies of intrathecal allogeneic MSCs.

Adverse events (person-time)	Total (%) *N* = 404	Our study (%) *N* = 146	Literature review (%) *N* = 258	*P*
Headache	10 (2.48)	3 (2.05)	7 (2.71)	0.940
Dizziness	8 (1.98)	2 (1.37)	6 (2.33)	0.771
Fever	8 (1.98)	5 (3.42)	3 (1.16)	0.232
Nausea	2 (0.50)	2 (1.37)	0 (0.00)	0.130
Pain at puncture site	7 (1.73)	6 (4.11)	1 (0.39)	0.018
Neck pain	1 (0.25)	1 (0.68)	0 (0.00)	0.361

**Table 8:** Clinical studies of intrathecal non-MSCs stem cells described in the literature.

Authors/country	Diagnosis	No. of treated patients	Stem cell type	Transplant type	Dose per injection	Injection frequency	Follow-up	Adverse events (person-time)
Callera and do Nascimento [40]/Brazil	SCI	10	Auto-BM HSCs	IT	100 × 10^6^	1	12 weeks	Uneventful
Kumar et al. [41]/India	SCI	297	Auto-BM MCs	IT	(3.66‐4.26) × 10^8^	1	18.4–20.5 months	Fever (95); headache (67); tingling sensation (68); spasm (1); neuropathic sensory symptoms (17)
Yang et al. [45]/China	Degenerative conditions^∗^	114	CBMCs	IT+IV	(1‐3) × 10^7^	4–5, weekly	4–5 weeks	Headache (19); fever (7); waist pain (5); shivering (3); vomiting (2); lower limb pain (2) (total: 592 person-time)
Sharma et al. [43]/India	MD SCI CP Miscellaneous	38 4 20 9	Auto-BM MCs	IT+IM	1 × 10^6^/kg	1	6–24 months	Headache (12); nausea (7); backache (10); vomiting (5)
Saito et al. [42]/Japan	SCI	5	Auto-BM MCs	IT	(3‐5) × 10^7^	1	6 months	None
Lv et al. [38]/China	Autism	23	CBMCs	IT+IV	(1‐2) × 10^6^	2-3, at 5–7-day intervals	6 months	Fever (3)
Mancias-Guerra et al. [44]/Mexico	CP	18	Auto-BM MCs	IT+IV	(4.38‐53.87) × 10^8^	1	6 months	Headache (2); vomiting (2); fever (1); stiff neck (1); lingual oedema (1); laryngeal stridor (1)
Zali et al. [46]/Iran	CP	12	Auto-BM PCs	IT	(6‐15.6) × 10^6^	1	6 months	Headache (5); nausea and vomiting (5); seizure (1); back pain (11)
Bansal et al. [47]/India	Autism	10	Auto-BM AC	IT	Not mentioned	1	24 months	None
Bansal et al. [48]/India	SCI	10	Auto-BM SCs	IT	Not mentioned	3, monthly	12 months	Spastic contraction (1); calf muscle pain (1); bronchoconstriction (1)
Liu et al. [35]/China	CP	34	Auto-BM MCs	IT	1 × 10^6^/kg	4, at 3–4-day intervals	12 months	Fever (3); nausea, vomiting, and headache (6)

Abbreviations: CP, cerebral palsy; MD, muscular dystrophy; SCI, spinal cord injury. ^∗^Included (no. of patients): paraplegia (42), ataxia (23), multiple sclerosis (19), amyotrophic lateral sclerosis (12), sequelae of cerebrovascular diseases (6), multiple system atrophy (4), motor neuron disease (2), cerebral palsy (1), nerve injury (brachial plexus) (1), traumatic brain injury sequelae (1), hypoxic-ischaemic encephalopathy sequelae (1), cervical spondylotic myelopathy (1), and optic nerve hypoplasia (1). Auto: autologous; AC: aspirate concentrate; BM: bone marrow; CBMCs: cord blood mononuclear cells; HSCs: haematopoietic stem cells; MCs: mononuclear cells; PCs: progenitor cells; SCs: stem cells; UCMSCs: umbilical cord mesenchymal stem cells.

**Table 9:** Comparison of adverse events between our study and previous studies of intrathecal non-MSCs stem cells.

Adverse events (person-time)	Total (%) *N* = 1389	Our study (%) *N* = 146	Literature review (%) *N* = 1241	*P*
Headache	114 (8.21)	3 (2.05)	111 (8.94)	0.007
Dizziness	2 (0.14)	2 (1.37)	0 (0.00)	0.011
Fever	114 (8.21)	5 (3.42)	109 (8.78)	0.026
Nausea	20 (1.44)	2 (1.37)	18 (1.45)	1.000
Pain at puncture site	32 (2.30)	6 (4.11)	26 (2.10)	0.125
Neck pain	1 (0.07)	1 (0.68)	0 (0.00)	0.105

**Table 10:** Comparison of adverse events among clinical studies of intrathecal autologous MSCs, allogeneic MSCs, and non-MSCs stem cells.

Adverse events (person-time)	Autologous MSCs (%) *N* = 1028	Allogeneic MSCs (%) *N* = 404	Non-MSCs stem cells (%) *N* = 1241
Headache	73 (7.10)	10 (2.48)^a^	111 (8.94)^b^
Dizziness	0 (0.00)	8 (1.98)^a^	0 (0.00)^b^
Fever	81 (7.88)	8 (1.98)^a^	109 (8.78)^b^
Nausea	10 (0.97)	2 (0.50)	18 (1.45)
Pain at puncture site	75 (7.30)	7 (1.73)^a^	26 (2.10)^a^
Neck pain	2 (0.19)	1 (0.25)	0 (0.00)

^a^ *P* < 0.05 compared with autologous MSCs. ^b^*P* < 0.05 compared with allogeneic MSCs.

## Data Availability

All data are provided in full in the Results and the necessary details can be provided by the corresponding author under request.

## Supplementary Materials

Supplementary Materials The procedures for preparing allogeneic BM-MSCs.
