# Supplementary material for: Safety and Feasibility of Repeated Intrathecal Allogeneic Bone Marrow-Derived Mesenchymal Stromal Cells in Patients with Neurological Diseases
Source: Stem Cells Int. 2019 Jul 25;2019:8421281. doi: 10.1155/2019/8421281 (PMC6683773; doi:10.1155/2019/8421281)
Supplement: Supplementary Materials — The procedures for preparing allogeneic BM-MSCs. [file 8421281.f1.pdf]

Supplementary Materials

Preparation of allogenic BM-MSCs

All procedures were carried out in accordance with the Ethics Committee of Sun Yat-Sen Memorial Hospital, Sun Yat-Sen University (Guangzhou, China). All healthy donors were informed the scientific contributions, possible risks and complications, the corresponding prevention and treating measures for bone marrow aspirations, and signed the informed consent form. Their bone marrow was aspirated through puncture of posterior superior iliac spine, and MSCs were purified from bone marrow samples using density gradient centrifugation. Cells are resuspended in Dulbecco's modified Eagle's medium (DMEM, GIBCO) with 10% fetal bovine serum (FBS, GIBCO) and then seeded into 25 cm^2 flasks and cultured at 37°C in 5% CO_2. After 48 hours, the suspended cells are removed by replacing medium. Medium is replaced every 3 days thereafter. When the culture reaches 80-90% confluence, MSCs are digested using 0.25% trypsin containing 0.53 mM EDTA and reseeded into 75 cm^2 flasks as passage 1. When the culture reaches 90% confluence, MSCs are digested using 0.25% trypsin containing 0.53 mM EDTA and divided into a 225 cm^2 flasks as passage 2. When the culture reaches 90% confluence, MSCs are digested using 0.25% trypsin containing 0.53 mM EDTA and divided into two 225 cm^2 flasks as passage 3 and MSCs were cultured in this way until passage 4. Then, MSCs are resuspended in freezing medium (90% DMEM + 10% DMSO) to a concentration of 1 x 10^6 cells/mL. Next, MSCs are removed into cryogenic storage vials. Finally, vials are placed in a 4°C refrigerator, and start the freezing procedure. Cells should be frozen slowly (-2°C/min from room temperature to 4°C, -1°C/min from 4°C to -15°C, -5°C/min from -15°C to -120°C). Then MSCs are transferred to liquid nitrogen storage. To conduct an experiment, the cryovial containing the frozen cells from liquid nitrogen storage is immediately placed into a 40°C water bath. When there is just a small bit of ice left in the vial, cells are removed into a centrifuge tube containing 10ml PBS. After centrifuging at 1500rpm for 5 minutes, MSCs are resuspended in DMEM and reseeded into a 225 cm^2 flask. When the culture reaches about 100% confluence in 225 cm^2 flasks, MSCs are washed with PBS for 3 times, digested with 0.25% trypsin and collected in a 50 ml tuble followed by centrifugation in 1500 rpm, 10 min. Then the supernatant was discarded and resuspended with normal saline (NS). After being washed in NS for four times, the final MSCs (5 $\times 10^6$ cells/mL NS) are resuspended in syringe prepared for transplantation. It should be noted that the medium of MSCs would be collected for bacterium, endotoxin, virus (EB, HBV and HIV) test before harvesting.

To detect the cell surface molecular, MSCs are digested with 0.25% trypsin and washed with phosphate-buffered saline (PBS). After incubation with specific antibodies (CD105-FITC, CD73-FITC, CD90-FITC, CD45-FITC, CD34-PE, CD14-PE, HLA-DR-PE, all antibodies are against human and purchased from BD Biosciences, Mountain View, California), MSCs are washed again and resuspended in PBS. Flow cytometry is performed using a BD Influx cell sorter (BD Biosciences),

and the stem cells are identified through a panel of antibodies as being CD45, CD34, CD14 and HLA-DR negative while CD105, CD73 and CD90 are positive.

Cell stemness is assessed by testing the ability of MSCs to differentiate into adipocytes, osteoblasts, and chondrocytes. To induce osteogenic differentiation, MSCs are cultured in 12-well plates (1.5×10^4 cells/cm^2) in 2 ml/well of DMEM (Invitrogen) medium with 10% fetal bovine serum (FBS, Gibco). At 80% confluence, medium is changed to osteogenic differentiation medium (OM) consisting of DMEM with 10% FBS, 50 mg/L ascorbic acid (Sigma), 10 mM-glycerophosphate (Sigma), and 100 nM dexamethasone (Sigma), of which half is replaced every three days until 21 days of induction. Alizarin red staining is used to confirm the osteogenic ability of MSCs. To induce chondrogenic differentiation, MSCs (2.5×10^5 cells) are centrifuged at 600 g for 5 minutes in a 15 ml conical tube. Then, MSCs are cultured with high-glucose DMEM with 1% ITS-Premix (Corning), 1 mM sodium pyruvate (Sigma), 50 mg/L ascorbic acid (Sigma), 10 ng/ml transforming growth factor-3 (R&D), and 100 nM dexamethasone (Sigma), which is replaced every three days. The total culture duration is 21 days, followed by toluidine blue staining. To induce adipogenic differentiation, MSCs are cultivated in 12-well plates (1.5×10^4 cells/cm^2). DMEM is supplemented with 10% FBS, 10 g/mL insulin (Sigma), 0.2 mM indomethacin (Sigma), 0.5 mM 3-isobutyl-1-methylxanthine (Sigma), and 1 M dexamethasone (Sigma) until day 21. Oil red O staining is performed after 4% paraformaldehyde fixation.
